# An Improved Method for the Isolation of Extrachromosomal DNA from the Pathogenic Free-Living Amoeba *Naegleria fowleri*

**DOI:** 10.3390/mps9040105

**Published:** 2026-07-07

**Authors:** Colm P. Roster, James C. Morris

**Affiliations:** Eukaryotic Pathogens Innovation Center, Department of Genetics and Biochemistry, Clemson University, Clemson, SC 29634, USA; croster@clemson.edu

**Keywords:** circular extrachromosomal ribosomal DNA element, episome, free-living pathogenic amoebae, *Naegleria fowleri*

## Abstract

The pathogenic free-living amoeba *Naegleria fowleri* is the cause of primary amebic meningoencephalitis (PAM), a central nervous system infection that is almost always lethal. One of the unusual features of the amoebae is the presence of ~4000 copies of a nucleolar-localized closed circular extrachromosomal ribosomal DNA element (CERE) that encodes the cell’s ribosomal RNA repertoire. It has historically been challenging to purify large quantities of CERE, limiting our understanding of the nucleic acid. Here, we describe a methodology for CERE purification that improves yield, reduces processing times, and maintains the integrity of the plasmid. This approach will enable the study of this unique DNA architecture, advancing our understanding of the pathobiology of the organism.

## 1. Introduction

*Naegleria fowleri* is an amphizoic pathogenic free-living amoeba and the etiologic agent of primary amebic meningoencephalitis (PAM), a nearly universally fatal central nervous system infection. Infection occurs when trophozoite-contaminated fresh water from sources such as lakes, ponds, and improperly purified tap water is forced up the nostrils. From there, trophozoites migrate to the cribriform plate and invade the brain. In the United States between 1962 and 2024, there have been 167 reported cases of PAM (Centers for Disease Control and Prevention). The outcomes of these infections have been very poor, resulting in only four survivors and a 97.6% mortality rate. However, PAM has a global distribution, with more than 380 cases reported worldwide [1]. This is certainly an underestimation, as new case reports have described large outbreaks in recent years, including the largest outbreak ever reported in Kerala, India, in 2024–2025 [2]. Although the clinical severity of the infection underscores the dire need for novel therapies, many aspects of *Naegleria* biology remain largely unexplored. This limits our understanding of virulence factors and pathogenic mechanisms, which in turn hinders the development of potential therapeutics.

A hallmark of the *Naegleria* genus that remains poorly characterized is the nucleolar-localized circular extrachromosomal ribosomal DNA element (CERE), which encodes all copies of the cell’s ribosomal DNA cistron [3,4]. CERE is commonly split into two sequences: the ribosomal sequence (RS), which includes the rDNA cistron, and the non-ribosomal sequence (NRS), a repeat-laden region hypothesized to contain necessary regulatory domains. Although each CERE only encodes a single copy of the rDNA cistron, *Naegleria* maintains 4000 copies per cell [4,5]. Eukaryotic ribosomal DNAs are ubiquitously maintained as multi-copy genes, including as extrachromosomal units [6,7]; however, the DNA architecture and high copy number are rare [8].

Our understanding of CERE biology in *N. fowleri* currently includes a handful of complete sequences. A putatively mapped origin of replication containing fragment and a homing endonuclease predicted to be associated with CERE have been characterized from the non-pathogenic *N. gruberi* [9]. For the pathogenic *N. fowleri,* little is known about the sequences that govern the replication machinery and origin licensing, rRNA transcription, and how conflicts between transcription and replication are resolved. This is, in part, due to challenges in isolating CERE. The non-ribosomal sequence (NRS) of CERE contains extensive repeats, which may alter its stability due to the formation of higher-order structures [10]. Further, as predators of other microbes, *Naegleria* species express abundant nucleases that could threaten CERE integrity during lysis [11]. Consequently, adapting the protocols used in other *Naegleria* species to *N. fowleri* has been unsuccessful.

Here, we describe a scalable methodology for the stable isolation of CERE from *N. fowleri* cell lysates. This protocol improves yield and reduces the technical demand of previously reported methods while maintaining plasmid integrity, enabling routine access to intact CERE. This approach provides a strong foundation for studying this unique DNA architecture in the biology of *N. fowleri*.

## 2. Experimental Design

The following protocol provides a detailed, stepwise procedure and required materials for standard *Naegleria fowleri* husbandry, cell lysis for CERE purification, assessment of CERE abundance and integrity, and downstream analysis of CERE dynamics. This protocol has enabled the complete sequencing and structural characterization of the unique extrachromosomal rDNA-bearing episome from *N. fowleri* TY strain amoeba.

### 2.1. Materials

1. Liver infusion broth (Becton Dickinson, Franklin Lakes, NJ, USA; Cat. No.: 226920);
2. Glucose (Macron Fine Chemicals/Avantor, Radnor, PA, USA; Cat. No.: 4912-06);
3. Sodium chloride (VWR, Radnor, PA, USA; Cat. No.: BDH9286);
4. Calcium chloride (VWR, Radnor, PA, USA; Cat. No.: BDH0224);
5. Magnesium sulfate (Thermo Fisher Scientific, Emeryville, CA, USA; Cat. No.: M65-500);
6. Heat-inactivated Fetal Bovine Serum (FBS) (Corning, Tewksbury, MA, USA; Cat. No.: 35-011-CV);
7. Penicillin-Streptomycin (Corning, Tewksbury, MA, USA; Cat. No.: 30-002-Cl);
8. Ice;
9. Phosphate-buffered saline (PBS) (Thermo Fisher Scientific, Emeryville, CA, USA; Cat. No.: 65-061-L);
10. Filter tips 1000 µL (VWR, Radnor, PA, USA; Cat. No.: 76322-154);
11. Filter tips 200 µL (MarathonLS, Norwood, MA, USA; Cat. No.: MLS-PTFS-200J);
12. Filter tips, 10 μL (VWR, Radnor, PA, USA; Cat. No.: 76322-132);
13. Filter Units, 1 L, 22 μm (Thermo Fisher Scientific, Emeryville, CA, USA; Cat. No.: 567-0020);
14. Tissue culture-treated T75 plug seal sterile culture flask (Thermo Fisher Scientific, Emeryville, CA, USA; Cat. No.: FB012938);
15. Treated, clear, 6-well plates (Avantor, Radnor, PA, USA; Cat. No.: 10062-892);
16. Sodium dodecyl sulfate (Thermo Fisher Scientific, Emeryville, CA, USA; Cat. No.: 28364);
17. Ethylenediamine tetraacetic acid (Thermo Fisher Scientific, Emeryville, CA, USA; Cat. No.: 5311-100);
18. Tris-HCl, pH 8 (VWR, Radnor, PA, USA; Cat. No.: BDH4502);
19. RNase A from bovine pancreas (Millipore Sigma, Burlington, MA, USA; Cat. No.: 10109142001);
20. Proteinase K (Worthington Biochemical Corporation, Lakewood, NJ, USA; Cat. No.: LS004248);
21. Glycogen (Millipore Sigma, Burlington, MA, USA; Cat. No.: G8751-5G);
22. Sodium Acetate (EMD Science, Burlington, MA, USA; Cat. No.: 7510);
23. Ethanol (Pharmaco, Brookfield, CT, USA; Cat. No.: UN1170);
24. DEPC-treated nuclease-free water (Thermo Fisher Scientific, Emeryville, CA, USA; Cat. No.: AM9906);
25. Agarose powder (Thermo Fisher Scientific, Emeryville, CA, USA; Cat. No.: BP160-500);
26. Boric acid (Thermo Fisher Scientific, Emeryville, CA, USA; Cat. No.: J67202.A1);
27. Sodium hydroxide (Thermo Fisher Scientific, Emeryville, CA, USA; Cat. No.: 5318-1);
28. Ethidium bromide (Thermo Fisher Scientific, Emeryville, CA, USA; Cat. No.: J66192.03);
29. Multiplate PCR 96-well plates (Bio-Rad, Hercules, CA, USA; Cat. No.: MLP9601);
30. Microseal “B” seals (Bio-Rad, Hercules, CA, USA; Cat. No.: MSB1001B);
31. pGEM-T Easy Vector System (Promega Corporation, Madison, WI, USA; Cat. No.: A1360);
32. Luna Universal qPCR Master Mix (New England Biolabs, Ipswich, MA, USA; Cat. No.: M3003S)
33. EvaGreen Dye, 20× in water (Biotum, Fremont, CA, USA; Cat. No.: #31000);
34. Monarch Spin DNA Gel Extraction Kit (New England BioLabs, Ipswich, MA, USA; Cat. No.: T1120L);
35. Bleach (Clorox, Oakland, CA, USA; Cat. No.: 044600100500);
36. 6× agarose loading buffer (New England BioLabs, Ipswich, MA, USA; Cat. No.: B7024S).

### 2.2. Equipment

1. Liquid nitrogen storage tank;
2. Biological safety cabinet;
3. Chemical hood;
4. Incubator;
5. Conical tube, 50 mL;
6. Hemocytometer with cover slips;
7. Pipettes;
8. 1.5 mL Microcentrifuge tubes;
9. Microwave oven;
10. Water bath;
11. NanoDrop Lite spectrophotometer (Thermo Fisher Scientific, Emeryville, CA, USA);
12. Inverted light microscope;
13. Tabletop refrigerated centrifuge (Eppendorf, Hamburg, Germany);
14. Fisherbrand AccuSpin Micro 17R tabletop refrigerated centrifuge with safety cups (Thermo Fisher Scientific, Emeryville, CA, USA);
15. CFX Opus 96 Real-Time PCR system (Bio-Rad, Hercules, CA, USA);
16. Owl Easycast agarose gel rig B1A-BP (Thermo Fisher Scientific, Emeryville, CA, USA);
17. Benchtop UV transilluminator (UVP/Jelight Company Inc., Irvine, CA, USA);
18. Power Supply (300 V) (VWR, Radnor, PA, USA).

## 3. Procedure

The approaches required for the culture and harvesting of *N. fowleri,* isolation of CERE, and downstream analysis are described below. This approach was designed to overcome preparative challenges related to CERE stability that we have encountered using more traditional DNA isolation methods. While the root causes of the challenges have not been resolved, we anticipate that lysis of the amoebae may liberate nucleases (potentially those related to the amoebae’s predatory nature) that cause rapid degradation of DNA.

### 3.1. Amoeba Growth and Passage (~1 Week)

**CRITICAL STEP:***N. fowleri* is considered a Risk Group 2 organism and should only be handled by trained personnel using appropriate containment. The work described below should be completed in a certified class II biosafety cabinet.

1. Thaw a frozen stock of *N. fowleri* TY strain (*Nf*TY, ATCC 30107) at room temperature.
2. Transfer the thawed sample using a 1000 µL filter-tipped pipette into a T75-treated plug seal culture flask containing Nelson’s complete medium (NCM; 0.17% liver infusion broth, 0.17% glucose, 0.012% sodium chloride, 0.0136% potassium phosphate monobasic, 0.0142% sodium phosphate dibasic, 0.0004% calcium chloride, 0.0002% magnesium sulfate, 10% heat-inactivated fetal bovine serum, 1% penicillin-streptomycin). Culture at 37 °C, 5% CO_2_ in a tissue culture incubator.
3. Monitor cells daily by microscope. When amoebae reach ~80% confluency, the cell culture should be passed to prevent overgrowth, encystment, or cell death.
4. For passage, place the near-confluent flask on ice for 20–30 min to release the cells from the bottom of the flask. Do not exceed one hour, as longer periods negatively impact amoebae health.
5. Collect the amoebae-containing medium by pipette and transfer to a sterile 50 mL conical tube. Centrifuge (3000× *g*, 4 °C, 5 min) to pellet the cells.

**CRITICAL STEP:** All centrifugations should be performed using centrifuge safety cups to contain the tubes and ensure aerosol containment.

**CRITICAL STEP:** Prior to removal of the sample tube from the safety cup, place the unopened centrifuge safety cup in the biosafety cabinet and disinfect the exterior with 70% ethanol. (Please note, this is not to eliminate amoebae, as they will be contained by the safety cup, but rather is to limit inadvertent introduction of contaminants from the centrifuge into the cabinet.) After removing the 50 mL conical tube from the safety cup in the cabinet, decontaminate the interior of the cup and exterior of the conical tube with 70% ethanol to eliminate amoebae that may have escaped the conical tube because of centrifugation-induced tube deformation and incomplete conical lid closure.

6. Remove the supernatant and resuspend the remaining cell pellet in 1 mL NCM.

**CRITICAL STEP:** Spent medium should be transferred to a waste collection vessel within the biosafety cabinet and decontaminated by adding 6% bleach to a final concentration of 0.5% for 24 h before disposal in approved hazardous waste containers. If a different starting concentration of bleach is used, adjust the amount added to reach a final concentration of 0.5% sodium hypochlorite.

7. Dilute the resuspension 1:100 in medium prior to loading onto a hemocytometer. Count the amoebae and then transfer the desired number of cells to a new treated tissue culture T75 flask containing 10 mL of NCM.
8. Return the flask to the growth incubator (37 °C, 5% CO_2_).

### 3.2. Lysis and Extraction (~7 h)

**Figure 1:**
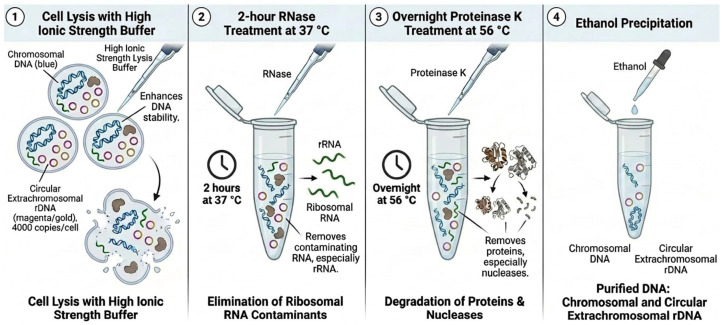
Schematic of optimized CERE purification.

1. Count amoebae as described above. The desired DNA mass and downstream applications will influence the number of cells needed.
2. Following centrifugation (3000× *g*, 4 °C, 5 min), resuspend cells in 300 μL of SNET buffer (1% SDS, 100 mM NaCl, 10 mM EDTA, 10 mM Tris-HCl, pH 8) and incubate at room temperature for 15 min. (NOTE: The salt concentration can be increased to decrease CERE degradation; however, it will limit the effectiveness of the RNase A treatment.) (See Figure 1 for a schematic of this preparation.)
3. Add RNase A to a final concentration of 100 μg/mL and incubate in a water bath (37 °C, 2 h).
4. Add Proteinase K to 100 μg/mL and incubate (56 °C, overnight).
5. Ethanol precipitate the DNA using the following approach:
  - Add glycogen to a final concentration of 20 μg/mL and then gently mix;
  - Add sodium acetate to a final concentration of 0.3 M, then mix gently;
  - Add 2.5 volumes of ice-cold absolute ethanol, then invert the tube gently to mix;
  - Incubate at −20 °C in a freezer for at least one hour (The sample can be left in 100% ethanol overnight without appreciable differences in recovery).
6. Centrifuge the sample (14,000× *g*, 4 °C, 20 min). Pour off the supernatant and wash the pellet with 1 mL of room temperature 70% ethanol. Centrifuge again (14,000× *g*, 4 °C, 20 min).
7. Air dry the pellet by inversion of the tube in a biosafety cabinet (to avoid contamination of the sample). When dry, resuspend in 150 μL of sterile water.
8. For further purification from contaminating genomic DNA (which may not be required for all applications, see below), run the sample on a 0.7% agarose gel and extract the band (see below).

### 3.3. Gel Analysis of Purified Product and Gel Purification

**CRITICAL STEP:** Preparation of molten agarose is a key step in pouring gels for analysis of purified CERE. Care should be taken to avoid overheating the agarose, as it can overflow the heating vessel if agitated. Additionally, ethidium bromide is a mutagen and a suspected carcinogen, so appropriate personal protective equipment, including nitrile gloves, a lab coat, and eye protection, is important during handling of the gels.

1. Make a 0.7% agarose gel by adding 0.21 g agarose to a microwave-safe container. Add 30 mL of 1 × TBE (generated by dilution of 3 mL 10× stock solution in 27 mL of water), mix, then microwave for ~20 s. Mix again, microwave for another 20 s, and repeat until the agarose is fully melted.
2. Allow the molten agarose to cool to ~50 °C and then add ethidium bromide (1 µg/mL final concentration), gently swirl to mix, and then pour into the gel casting tray. Allow the gel to remain undisturbed until the agarose has solidified (~20 min) and then gently remove the well combs.
3. While the gel is cooling, prepare the samples by mixing with sufficient 6× agarose loading buffer to yield a final 1× concentration.
4. Cover the solidified gel in 1× SB buffer and load the gel by pipetting with loading buffer into the wells. Resolve the DNA at 100 V, 50 mA for 2 h.
5. Visualize the DNA on the transilluminator.

**CRITICAL STEP:** The UV light emitted by the transilluminator can damage the skin and eyes. Wear appropriate protection, including a face shield, lab coat, and nitrile gloves, to limit exposure.

6. To gel purify samples, transfer the gel to a transilluminator and carefully excise the DNA from the agarose gel using a clean razor blade or spatula, limiting the amount of excess gel material associated with the DNA band through precise cutting. (Please note, this process may introduce thymidine dimers in the DNA, so limit exposure to the sample, if possible. For needs where dimers and other DNA damage should be avoided (for example, sequencing), we recommend only using a fraction of the CERE preparation for validation of purity by agarose gel and using the remaining for downstream applications.)
7. Using the Monarch Spin Gel Extraction kit, purify the isolated DNA and quantify using the NanoDrop spectrophotometer. Store the DNA at −20 °C in the elution buffer provided with the kit until needed for downstream applications.

### 3.4. CERE Analysis by qPCR

1. For qPCR, primers targeting the chromosomal glucose-6-phosphate 1-dehydrogenase gene (G6PD, NfTy_000310) and CERE-specific non-ribosomal sequence (NRS) were used to score the CERE copy number.
  The following primers were used:
  G6PDforward: 5′CGCGAACGTTATTCTTGAACC3′
  G6PDreverse: 5′TAGCCTCCTCTTCCTTCTACTC3′
  CERENRSforward: 5′TTGGTGCACAGGTGACTTATAG3′
  CERENRSreverse: 5′CCGTCACACCCTTATCCTAAAC3′
2. To validate the performance of these primers, cellular DNA was purified from 5 × 10^6^ *Nf*TY amoeba using the described CERE extraction without gel extraction. Cellular DNA dilutions were made from 10 ng to 1 pg in 50 µL of water, and 5 µL of each dilution was used in qPCR reactions (20 μL) set up in a 96-well plate on ice. Other reaction components include 10 μL 2× Luna qPCR MasterMix, 0.5 μM of each primer (0.5 μL of 10 μM stocks), EvaGreen (1 μL of 20× stock), and water to 20 μL. Plates were sealed and transferred to a BioRad CFX Opus 96 Real-Time Systems instrument. The qPCR program included an initial denaturation (95 °C, 60 s), followed by 40 cycles of denaturation (95 °C, 15 s) and extension (60 °C, 30 s). A melt curve was also performed for all samples to test primer specificity. NCM Primer pair validation was assessed by plotting Cq values on the y-axis and the log(DNA concentration) on the x-axis to score the linear relationship between template concentration and Cq values.
3. qPCR experiments were run using the same parameters and approach with optimized primers to score CERE abundance.

**CRITICAL STEP:** For standard qPCR reactions, template DNA should not exceed 100 ng per reaction.

### 3.5. CERE Sequencing

1. To sequence CERE, the extrachromosomal element was purified as described above from 5 × 10^6^ *N. fowleri* TY strain cells.
2. Portions of CERE were amplified using PCR. Primers (below) were added to a master mix containing 2× GoTaq Green Master Mix, 250 nM primers, and 20 ng of template. PCR programs included an initial denaturation (95 °C, 2 min), 25 cycles of denaturation (95 °C, 15 s), a two-step annealing protocol (55 °C, 30 s; 45 °C, 30 s), and a low-temperature extension (65 °C, 1 min/kilobase amplicon). The complete assembled sequence can be found in Supplemental Data S1.

**CRITICAL STEP:** Standard PCR conditions can be used to amplify CERE. However, the NRS is a repeat-laden sequence, so the modified annealing and extension temperatures are key to allow for improved on-target amplification.

The following primers were used:

NheI.NfCERE.1F: 5′GCTAGCTACCTGGTTGATCCTGCCAG3′

NheI.NfCERE.1R: 5′GCTAGCAAATGATCCCTACGCAGGTTC3′

NheI.NfCERE.2F: 5′GCTAGCGGGAAACCAGTTAAATCTATTCG3′

NheI.NfCERE.2R: 5′GCTAGCTCCCCTTATTAATATGCTTAAATCC3′

NheI.NfCERE.3F: 5′GCTAGCCTAGCCTCTAATGTGAGAGG3′

NheI.NfCERE.3R: 5′GCTAGCGATCTAGTGTTGGCTATTAAGG3′

NheI.NfCERE.4F: 5′GCTAGCATTAGAGCCATGTGCGTGG3′

NheI.NfCERE.4R: 5′GCTAGCTATCAAGTTGGGGACTTTACG3′

NheI.NfCERE.5F: 5′GCTAGCCGAGACAAGGCTTCTTGTCG3′

NheI.NfCERE.5R: 5′GCTAGCCATTAAAGTCCTGCGTACTCC3′

NheI.NfCERE.6F: 5′GCTAGCGGACTTTAATGATAAGGTCACCAAGG3′

NheI.NfCERE.6R: 5′GCTAGCTCCGCAATTTCGTTCGAATCCAATTCC3′

NheI.NfCERE.7F: 5′GCTAGCATTTTTATTTCCTGAACGGCCATTCAGGAAGGG3′

NheI.NfCERE.7R: 5′GCTAGCTCGCTTAATATTTTTACTAAGTAG3′

NheI.NfCERE.8F: 5′GCTAGCCGAACGACCCAAAAAATTTAACG3′

NheI.NfCERE.8R: 5′GCTAGCGTCATTATAGTGGCGTGAACC3′

3. PCR products were gel-extracted using the Monarch Spin Gel Extraction kit. Gel-extracted bands were ethanol precipitated to remove any remaining salts and then sent to Plasmidsaurus (Louisville, KY, USA) for linear amplicon sequencing. Overlapping sequences were assembled into a contig in SnapGene (San Diego, CA, USA).

## 4. Expected Results

### 4.1. Extraction Methods Are Scalable and Produce Stable CERE

**Figure 2:**
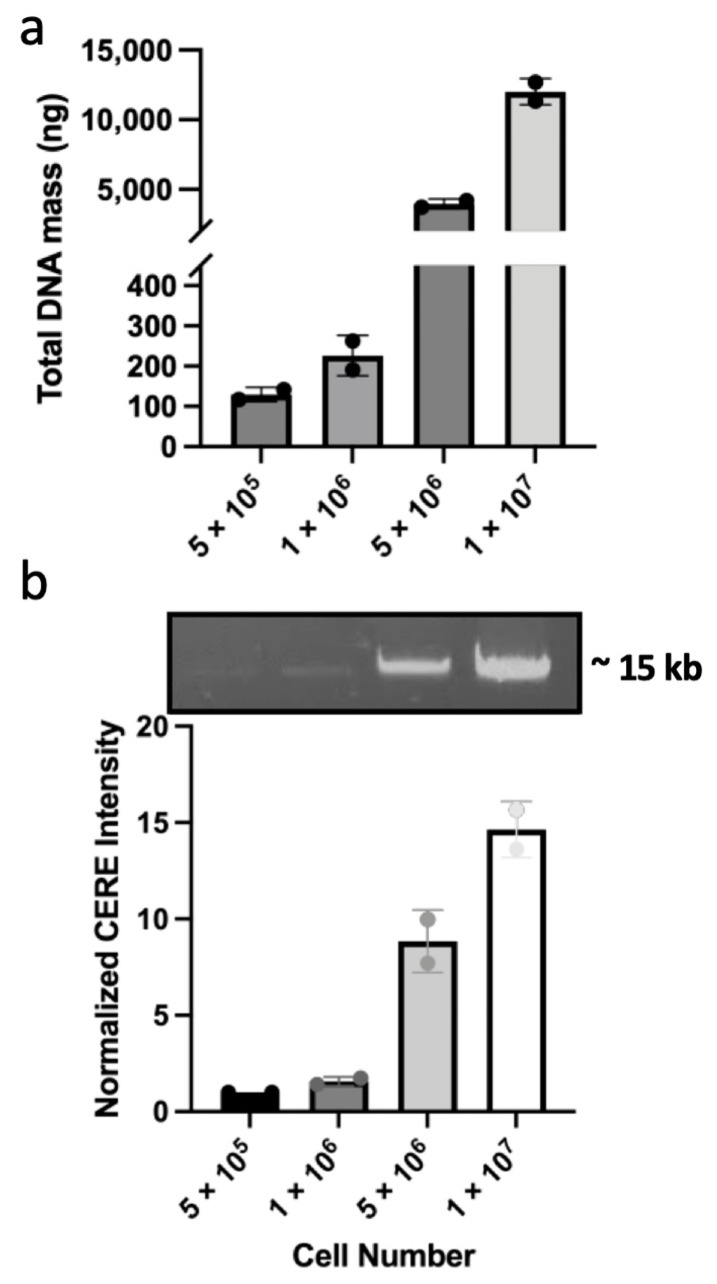
Recovery of CERE is influenced by the starting cell number. (**a**) Total CERE mass extracted using a high ionic strength SNET buffer. Total DNA mass was calculated by assessing concentration by spectrophotometry. (**b**) CERE is extracted linearly with respect to cell equivalents. CERE was extracted and analyzed by agarose gel electrophoresis, followed by densitometry that measured the intensity of the CERE band after subtraction of background pixel intensity. CERE values were normalized to the value generated from 5 × 10^5^ cells. The intensities for two biological replicates were measured and plotted in GraphPad Prism (version 10.6.1(799)) and an ordinary one-way ANOVA with Tukey’s multiple comparison and single-pooled variance was performed (5 × 10^5^ vs. 1 × 10^6^: *p* = 0.9478, 5 × 10^5^ vs. 5 × 10^6^: *p* = 0.0068, 5 × 10^5^ vs. 1 × 10^7^: *p* = 0.0008; 1 × 10^6^ vs. 5 × 10^6^: *p* = 0.0090, 1 × 10^6^ vs. 1 × 10^7^: *p* = 0.0010; 5 × 10^6^ vs. 1 × 10^7^: *p* = 0.0202).

Previous CERE extraction techniques have faced limitations regarding scalability due to their repurposing of silica columns. The methods described here are scalable, as total DNA mass increases linearly with starting cell number up to 1 × 10^7^ cells (Figure 2a). Similarly, CERE recovery increases relative to starting cell number, as determined by densitometry following agarose gel electrophoresis (Figure 2b). The single species of CERE noted during gel electrophoresis highlights that CERE integrity is largely maintained during extraction (Supplemental Figure S1).

### 4.2. CERE Relative Abundance Is Maintained During Extraction for Lower Cell Numbers

**Figure 3:**
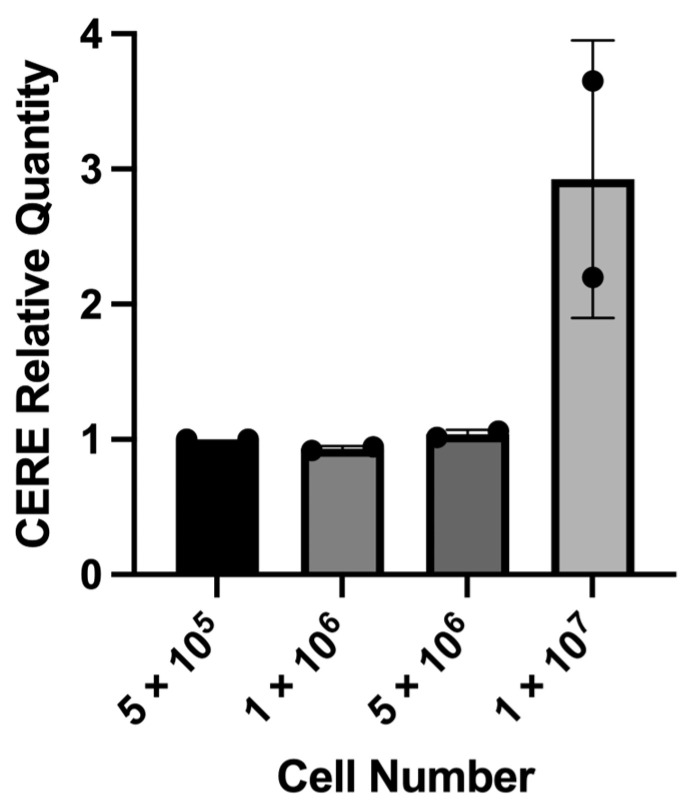
CERE abundance is proportional to chromosomal DNA as scored by qPCR. qPCR reactions were performed using primers to the CERE NRS and G6PD, with the latter serving as a chromosomal reference gene. Samples for each cell number were tested in biological duplicate and technical triplicate. CFX Maestro software was used to calculate the relative abundance of CERE to chromosomal DNA for each starting cell number, which was normalized to the values generated for 5 × 10^5^ cells. Normalized values were plotted in GraphPad Prism (version 10.6.1(799)) and an ordinary one-way ANOVA with Tukey’s multiple comparison and single-pooled variance was performed (5 × 10^5^ vs. 1 × 10^6^: *p* = 0.9995, 5 × 10^5^ vs. 5 × 10^6^: *p* > 0.9990, 5 × 10^5^ vs. 1 × 10^7^: *p* = 0.0922; 1 × 10^6^ vs. 5 × 10^6^: *p* = 0.9980, 1 × 10^6^ vs. 1 × 10^7^: *p* = 0.0840; 5 × 10^6^ vs. 1 × 10^7^: *p* = 0.0973).

To assess the viability of this protocol to explore CERE copy number dynamics, we validated that analysis of CERE by qPCR quantification was not impacted by the new extraction method. The relative abundance of CERE to chromosomal DNA was maintained using different cell numbers for CERE preparation (Figure 3). A slight increase in CERE abundance relative to genome abundance was noted when 1 × 10^7^ amoebae were used, possibly because of preferential precipitation of the biomolecule at high concentrations.

## 5. Discussion

*Naegleria fowleri* infection is nearly universally fatal. Improving prognosis has remained challenging for two primary reasons. First, an earlier diagnosis is likely critical to improving patient outcomes. Second, the lack of efficacious treatment options is likely a large player in the high mortality rate of PAM. Given that CERE is a *Naegleria*-specific biomolecule that encodes the sole template for ribosomal RNAs, it is positioned to be a basis for tackling both of the variables confounding successful treatment. Here, we describe a protocol that allows the extraction of large amounts of CERE from cell lysate. This approach will allow further assessment of CERE as a platform to improve diagnostic measures while enabling studies to explore CERE maintenance and replication as potential therapeutic targets.

CERE extraction methodologies developed for other *Naegleria* species that have relied on the use of commercial kits or liquid–liquid DNA extraction methods have proven ineffective for *N. fowleri* [9,12]. The lack of scalable isolation approaches for *N. fowleri* CERE has impeded CERE-specific research, limiting our current knowledge to a few sequenced specimens and characterization of several putatively mapped origins of replication [5,9,13,14]. The isolation approach described here will allow for improved understanding of CERE-related processes.

CERE is unique to the *Naegleria* genus, and because of its high copy number, other researchers have explored its use as a diagnostic tool by scoring the internal transcribed spacer sequences of the rDNA cistron [15]. The extrachromosomal localization of CERE has remained puzzling, as most eukaryotes maintain chromosomal distribution of rRNA DNAs. It has been hypothesized that this extrachromosomal localization allows for amplification of rDNA copies as a response to different translational demands that are environment dependent. This has been observed in other systems, where depleting rDNA copy number incurs a sharp fitness cost [16].

Beyond being an important biomolecule for a human pathogen, CERE represents a promising system for studying eukaryotic DNA replication and maintenance. As CERE replicates autonomously from chromosomal DNA and each molecule contains its own origin of replication, CERE has the potential to become a tractable model for studying origin licensing. Additionally, we anticipate that the study of CERE will provide novel insights into DNA-protein interactions during replication and the evolutionary advantages of differential rDNA copy number [9].

Ribosome biogenesis is necessary for cell health and all cellular functions. Inhibitors to rRNA transcription have been explored as therapeutics for cancer and other protozoan parasites [17,18]. Inhibitors that target CERE-related processes, such as ribosome biogenesis, have yet to be explored as potential *N. fowleri* treatment options. We hypothesize that agents that either directly inhibit rRNA transcription or decrease CERE copy number may represent highly potent and specific therapeutic options for this devastating disease.

## Data Availability

The original contributions presented in this study are included in the article/supplementary material. Further inquiries can be directed to the corresponding author.

## Supplementary Materials

The following supporting information can be downloaded at: https://www.mdpi.com/article/10.3390/mps9040105/s1, Data S1: Complete assembled sequence for the *N. fowleri* TY strain CERE; Figure S1: Agarose gel image of purified CERE; DNA was extracted following the described protocol and equal volumes (20 µL) were resolved on a 0.7% agarose gel (100 V, 50 mA, 2 h). The gel was then stained with ethidium bromide (1 µg/mL final concentration) and imaged. Size was estimated using the 1 kb extend DNA ladder (New England Biolabs, Ipswich, MA, USA) and ImageJ (Version 1.54t 16 May 2026) was used to assess pixel density over background to assess relative CERE abundance.

## Abbreviations

The following abbreviations are used in this manuscript:

CERE	Circular extrachromosomal ribosomal DNA element
PAM	Primary amoebic meningoencephalitis
NRS	Non-ribosomal sequence
NCM	Nelson’s complete medium
