# Supplementary material for: An Improved Method for the Isolation of Extrachromosomal DNA from the Pathogenic Free-Living Amoeba Naegleria fowleri
Source: Methods Protoc. 2026 Jul 7;9(4):105. doi: 10.3390/mps9040105 (PMC13398248; doi:10.3390/mps9040105)
Supplement: Supplementary file 1 [file mps-09-00105-s001.zip › mps-4355213-supplementary Data S1.pdf]

>N. fowleri TY strain circular extrachromosomal ribosomal DNA element (Nf.TY CERE)
TACCTGGTTGATCCTGCCAGTACTATATGCTTGTCTCAAAGCCTAAGCCATGCAAATGTAAGATCAGTCATAT
GTGGTTC
ACGCCACTATAATGACAGTCTGTGGAAGGCTCATTATAACAGTTATACACCTAGCCACTGGAAAGTTTACAAG
GATACCA
CCGTTAACTGCAGCGATAATACTTGTTCCCTTCGGGGTGGTAGTAGTATTTGTGCTGAAACCTAGCTATTGTA
ACTCAGT
TTCTCTGGGTATAGCAATATATTCAGGGGAGCTGGGCATCGACCGCTAGCAGGTGCCCGCAAGGGCGCGGGAA
AGTGAGC
TAACAAGGTTTTCATAAGGCCTTTCAGGTTTGCTTTTTCTAGTGGCCAGGCAGAGGTGTTTCTTACCTATCAG
CTCGTTG
TTTGTTTAAAGGACAAACAAGGCTTTGACGGGTACGGGGAATCAGTGTTCGATTCCGGAGAGGGAGCCTGAGA
GATCGCT
ACCACATCTAAGGACGGCAGCAGGCGCGCAAATTACCCAATCCCAACACGGGGAGGTAGTGACAAGATATAGT
GTCTCAT
CACCTTCGGTGGTGAGGTATTATCATTTGATAATTTTCCATGATTTGGGTGTAGATAACCCTTAGAGCAGCCA
TTGGAGG
AAAAGTCTGGTGCCAGCACCCGCGGTAATTCCAGCTCCAAGAGCGTATATTAATACTGCTGTAGTTAAAACGC
CCGTAGT
AAACCTAAGAGTGTTTACGGGGTAACTAGTTCCATCAGGAGATGGTTGGAGGGAGTTTATAACTCCCTCTTGC
CTACTCT
TGGTGAACTTTAGCTGGACAGGACTTCTGGTCCTCGTCTGGCAGTTGCCTCGTATTCACTCGTCCACGGTTCA
TCCGTGA
GGCCCCTTTGGGTTGCAACTGTAAATAAATCGTTGTGCTTAAAGCGGGCTATGATACTCTGCCAGAGCGATTT
AGCATGG
GACTGCAGAGTAGCTGTATTTGAGCGAAGGTTGGACTTCGGTCTGACTGTAGCTTGGTACAGCGCTTGTAATG
GAGCTCA
GGGTGAGGCCCCGGGTACCATGAGGCTAGAGGTGAAATTCTGAGACCCTCATGTGACCAACTAAGGCGAAAGC
TGTCGTG
GGCCACCACAAGCTCGTCTATCAGGGACGAAAGTTGGGTTATCGAAGACGATTAGATACCGTCGTAGTCCCAA
CTATAAA
CGATACCAACCGAGTATTTGGGAAGACACTATCCCAGCGATCTTCTCAGAACTCAAGGGAAACCTTAAGTCTT
TGGGTTC
TGGGGGGAGTATAGTCGCAAGACCGAAACTTAAAGGAATTGACGGAAAGGCACCACCAGGAGTGGAGTCTGCG
GCTTAAT
TCGACTCAACACGGGGAAACTCACCAGGTCAGGACACAAGTTTGATTGACAGGTTAATAGCCCTTTCTTGATT
GTGTGGT
GGGTAGTGCATGGCCGTTTCCAGTTCGTGGAGTGATCTGTCTTGTTAATTCAGATAACGAACGAGACCTAAGC
CTTTAAC
TAGCCGTAGGCCTCTTCCTTCGGGGAGGGGTTAGTTTGTCGGGACTGGCTTTTAGCCTGTTCCAAAACCTACG
TGACTTT
TGTCAGCTTCTTAAAGGGACTTCATTCGTTCTTGTAGAATGAGGAAGATTTAGGCCATAACAGGTCTGTGATG
CTCTTAG
ATGTCCTGGGCTGCACGCGTACTACAATAACGGTACCAGCGAGCGCTATGGTTTTTTAACCCCTTATCCTAAT
AGGATTG
GGTAAACTTTTCAAACACCGTTATGACAGGGATCGAGGATTGGAACATCCTCGTGAACGAGGAATTCCTAGTA
AGCGTGG
TTCATCATACCACATTGATTACGTCCCTGCCTTTTGTACACACCGCCCGTCGCTCCTACCGATGGGACGAAGA
GATGAAC
CTGGCGGACCAAACCGTAAGGTGAGGGAAACCAGTTAAATCTATTCGTCTGTAGGAAGGAAAAGTCGTAACAA
GGTCTTC
GTAGGTGAACCTGCGTAGGGATCATTTATGGTAAAAAAGGTGAAAACCTTTTTTTATGGTAAAAAAGGTGTAT
GGTAAAA

AAGGTGAAAACCTTTTTTCCATTTACAAAAAATAACTCTGTGCAATGGAGCACACGGCTCGTGTATCGATGAA
GCCCGCG
GCAAAAAGCGATATGTAATGAGATTCGTTAGCCTCGAGATTCATCAAATTGGTGAACACAGTCTGGACCTCGC
AAGAGGT
ACTTACGTTAGAGTGCTAGTTTTATATCAATTGATACTGGTAAAAGGTGTATTTAATCAATAGATTTTTACGC
CCTAGCT
GGTTATGCCGGATTCTCTTTGAGAAAACCGGATTGTCCCATTTGAAATTTTTTCAAATGGTCAATCTTTTATT
TAACTAG
CCTCTAATGTGAGAGGGTACCCCCTGGATTTAAGCATATTAATAAGGGGAGGAAAAGAAATCAACAGAGATTC
TCCTAGT
ACCTGCGAGGGAAAAGAGAAAAGCCCATCCTGAATCCAAGGTTTTACCTTGGAGTTGTAGGGTGAGGATGCTA
TACTTTG
ATCTGAGAATCGTGAAATTACCTGTAAAGGTATTCACAAGTGAATCCATAGAGGGTATTAGGCCCGTTAGCGA
TTCATTC
AGGTCTGTACGGTATGGTATCTACGAGTAGGATTGTTTGATAGTACAATCTGAATATAGGGGATAAACTTCCT
CTAAGGC
TAAATATAATGCACGAGACCGTTAGTAAACAAGTACCGTGAGGGAACGTTGAAAAGTACTCTGGTAAGAGGGT
GAAAAGA
GTGTGAAATTGCATAGATTTAAGCGTAACCAAGTATCGACTCTTGTAGTGTTCTTTTTGGTTCACTATAGGTA
TCCAACA
ACTCTGTTCTTGTTTTAATAAACGGGAATTGGGCTACACATGCTAATAATGCCGTTGTAGATGAGTTGTACAG
ATGAAGG
TCTTGTTGCTGTGTTCTTGCTTCACGGCTTGAATGCGGTACCGGGGTTGTAGTCTGTATGATTTGTCGCAAAT
AACCGGT
CCTTTAGCATATTGTTGGTATGGTATTTGGGAACGTCCCGTCTTGAAACACGGACCAAGGAGTCTAGCATATC
TGCAAGC
CGTAGCAATTTTAATTGTGAAGGCGTAATGAAAGTGTATTGTGTTAACAGCACAAACCGGGCCTTTAGGTCTG
AGTTAGA
GTAAATATGCTAGTACCCGAAAGGTGATGAACTATGCCCGGACTGGTTGAAGTCAGGAGAAATCCTGATGGAG
GACCTCC
CGCGGTGCTGACGTGCAAATCGCTCGTCCGAGCTGGGTATAGGAGCGAAAGACTCATCGAATCGCCTAGTAGC
TGGTTCC
TGCCGAAATTTCCCTCAGGATAGCGGAGATCTTCAAATAGTTTTGCCAGGTAAAGCTAATGATTAGAGATCTT
GGAGGTT
GATAGCCTTCAATCTATTCTCAAACTTTAAATGGGCAATATCCAGAACTTACTTAACGGAAGTGCTGGTTTAA
TAAGGAT
CTCTAGTGGGCCGTGGTTTGGTAAGCAGATCAGGTAAAGCGGGTTCAACCGTAAACAATAAGGAAAGGCGTCC
AATACCG
GTTTAATACCCACTAAAGGGTGTTAATTCATGAAGACAGCGGGACGGTGGCCATGGAAGTCGGTATCCGCTAA
GGAGTGT
GTAACAACTCACCCGCCGAATGAATTAGCCCCGAAAATGGATGACGGCTAAGCCGGTAGCCGATCCTTAGTCT
CTCTTCT
AGATTTCTTGAAGAGAGGGTAGGCAGGCGTGATGGTTGCTGCGAAGCTCTTGGGTGACTGGGAGTGGAGCGGC
CATCAGT
GCAGATCTTGGTAGTAGTAGCAACCATTCTTACGAGCTTCAATCCCCATAGGCAACTATGGGTTTAAAAGCTT
TGAAGGT
CGTAGCGGACCAGGTTTCCCTACCTCTGGCGCTCATGTAGGGGTTAGTCGGTCCTAACTTGGTGGTTAACACT
AGGAATA
GTCCTAAAGTAGGAATTACCAAGGAAAGGGAATCAGGTTAATATTCCTGAACCTAAGTCATGGAGGATACTTT
CGGGTAT
CAAGTCTAACGCGGCGACGCGGAGTGTGGATTAGAGCCATGTGCGTGGAGCTCCGGTAGGGTTATCTCTTCTC
CTTAATA
GCCAACACTAGATCACGAGGCTTTGGCTGAGTGGCTGGTGGCGAGTAACAGAGGAGTTGCGTGCAGCAGAGAT
TCTGTTA

TTTGCGGGTTAGAAGGGCAAATCTTTATGATTTGTGAGGGTCTTCGCGTGCATAAGTTAAAATCTAATTCAGC
TCTGGAC
TCAATCCTGTGACTTAGTCCGTACCGAATTCCGCATCAGGTGTCCGAGGCTAGCAGCCTCTGACCTATTAGAG
CAATGTA
GGTAAGGGAAGTCGGCAAAATAGACTTGTTGCTTCGGTGGATAGGTTGGCTCTTGTAGTGATGTTCATTGGCT
ACTACAT
GAAGTCTGGCGCCGCAAGGTCATGCAGGTAGGGGTGGACTGGGCTCTCGGGTCCAGGTAAGCCTCTATTTGCT
GCTGGAT
GTAGTGTAGTAGCAGGCTTAGCAAACTTGTTTGCGAGGTCGCACAATGGACGTTTAGTCACATTACGAGAACT
CATGGAG
TCTAGGGGAATCCAACTGTTTAATTAAAACAAAGCAAGGTGTCCGAATCGTAACGGTCCTGCACACCTTGTGA
TTTCTGC
CCAGTGCTCTGAATGTCAAAGTGAAGAGATTCGATCAAGCACGGGTTAACGGCGGGAGTAACTATGACTCTCT
TAAGGTA
GCCAAATGCCTCGTCATTTAATTTGTGACGCGCATGAATGGAACAATGAGATTCCCACTGTCCCTACCTACTG
TCTAGCG
AACCTACAGCCAAGGGAACGGGCTTGGGTTGGTTAGCGGGGAAAGAAGACCCTGTTGAGTTTGACTCTAGTCT
GGCGTTG
TAGAATAACGAAGGAGGTATAGTATAGGTGGGAGCTTCGGCGACAATGTAATACCACTACTCCTTATTGTTAT
TCTACTA
ACTTCATAAGAGAACGAGTGTAGCGATACACTTTTTTGGTAAAAAGATACCGAGTTCATGATCGATGGAGAAC
ATCGCCA
GGTGGGGAGTTTGGCTGGGGCGGCACATCTGTTAAAAGATAACGCAGGTGTACAAAGGTCATCACAACAAGGT
CAGAAAC
CTTGTGTTGCGGATAAGGCCAATAGATGGCTTGACTAATACCTCCAGTAAAGGTATTAGATGCGAAAGCATGT
CCCAGCG
ATCCTTTAGTTTCTGTAGCAATACAGGATCTCAAAGTTTGAGGCTAGAGGTGTCAGAAAAAATTACCATAGGGA
TAACTGG
CTTGTGGCCGCCAAGCGTTCATAGCGACGTGGCTTTTTGATCCTTCGATGTCGGCTCTTCCTATCATTGCGAA
GCAGAAT
TCGCAAAGTGTTGGATTGTTCACCCACTAACAGGGAACGTGAGCTGGGTTTAGACCGTCGTGAGACAGGTTAG
TTTTACC
CTACTAACCAATTTCGGTGCCAGTAGTAAATCTTAGTACGAGAGGAACAGCTTTGCCGTGCATTTAGTCGCAG
ATCCGTT
TGAAAGGATGTGATTTTTCGCCACGCACGTAGATGTATAACTGAAAGCCTCTAAGTTATGAGTCTTACTGTGA
TGCTGAG
TACGGCTCGCTTCTAACGAGTGAAAATAACAATTAACTCATTTTGGTGGTAATCAAAAAGAAGTGGGCCTTAT
CAAATAA
GGAGACCACTTATGACCCCTCTTTGAAGGGACCTACTGTCGTACCTGGGCGAGACAAGGCTTCTTGTCGACTC
ACTGACG
CTCGTAAAGTCCCCAACTTGATAAAGGTTTTTGGGGAGTTTTCGAACTTCAACGACAATTTCTTCCAAACTTG
TCTTTGT
TATGTGATTAAAGTTCCTGATCAGTTCTTTGGTCATGTATCAAGGACAAGTACTCTTTTTTTTTGTTGACTTG
AGGGTCT
GATCAACTTTCTTGTCTTCAAAAAGATTAGAGTGTTGGAAAAAAAATATCTGTTTTATTACAGATTTTTTTTTA
ACAAAAT
TAAATAATTAGAATTTTAAAAATTAGAATATACAAGACGTATTTCAAGAATTTTTTTACTTTGAATCGACTTG
TAATTTT
TTTTAAAAAATTTTGTTTCTTGTCTTCAAAAAGATTAGAGTGTTGGAAAAAAAATATCTGTTTTATTACAGATT
TTTTTTT
AACAAAATTAAATAATTAGAATTTTAAAAATTAGAATATACAAGACGTATTTCAAGAATTTTTTTACTTTGAA
TCGACTT
GTAATTTTTTTTAAAAAATTTTGTTTCTTGTCTTCAAAAAGATTAGAGTGTTGGAAAAAAAATATCTGTTTTAT
TACAGAT

TTTTTTTTAACAAAATTAAATAATTAGAATTTTAAAAATTAGAATATACAAGACGTATTTCAAGAATTTTTTT
ACTTTGA
ATCGACTTGTAATTTTTTTTAAAAAATTTTGATCGGGGTTGATACAACAAAAATTGTATCAAGTCATGCAAAG
GATGGTT
ACCTTTGCAAATATGGCGACACTGTCAAATTGCCGGAAAATCCTGTTAAGTCTATAGTACCGCTCTAGTACCG
AAAGGTC
TGACTAGTTGCACCATAGAGAAATCTATGGGTATGGTAAGAATCTATGGAATAAGGATAATCGGCAGCCAAGT
CCTAAAC
CATATTTTAAAGATATGGCATGGATGCAGTTCAGAGACTAAATGTCAGTGGGTCATTCATATATGAGAATGGC
TTAAGAT
ATAGTCCGCCCCACCCGAGAGGGTGCTCTAGGAAGAAGGAGTCTAACCAGCTCCCCGAGCCTAGAGAGGTCAA
ATCTCTT
TAAGAGATGGATTGACTGGGATAAAGTTTCAAACCCTGGAAACGTTCCCAAATAACTACCTATTTGTTTCCTA
ATAACAT
TGTATATATAATAATAATGGCAAAACTTGTAAAACGTAAAAACGTAATAGCTAGACGTGTGAACAAACATCTA
CCTGTCG
TCCTGTTAGGATCAAGATGTCACATACCACCGAGATATCTAAGAAAACCTCCAAAACTTAAAAAACCTCCAGC
CCCACTC
AAAGATGAACGAGATTGGATCTATCCAAACACATATACGGTACAAGATTTTTTAATGTTTTATAAAGTACTCG
ATGCTGT
AGCTAAAGTTTACGAAAGACCCCCACATAAAGAAGAGAATTACCCTCCTGTAAATAATGAAGATTCTAGTTAT
CTCTATA
CACATTTGAAATATATAATTTACAAAGAAAGTAAAACAAAAGGATGCTGCATTTTTTCAAAGTATAAGAAAGG
TTCTAGA
ACTAGAACAGAAAATGTTTGTTTCAGATTACCAATGCCTATTATTAAATGCATTAGAAAACTAAATCTTAATT
GGAGAAC
TGAAATCAAACAACCTTCTGTTCCAGCTACTTGGATTGTATGTTGGTTAAACAGAATTTGGCCAGATACAAGT
ATAAATC
CTTATCAAGGACCATATGTATTGTGCAATTCTTGTAAAAATATTGGATGTCTCAATCCAAAGCATCTCTCCTG
GAGAAAT
TAAACAACAATAAATCTTTTTTTTAAAAAATTTTTAATAAATTAGTTTTCAAGAAATTAATACAATACAAAAC
CAATAAC
ACTCTAATCGTTTTTTTCTCAGTTAGACTTATCCACCAAATTTAAGAAATCAAATTCTCAAAATTAATTTCTT
TTCTAGA
CAAAAAATCTAGACAGCAAAATCTGTAAAAACTATGTTAATGAGAAATATATTAAATATAATAAGTTCTATCT
TTCAATT
AAAAGAATGTAATTAACGATAAAAAAATTTTTCTCCGACTTGTAATTTTTTTTCAAAATTTTTAAGTAAATAGT
TTTCAAT
AAATAATAAAAAAATTTACTATTAATTGTATTAAGTTGTTATTTAATAAAATCAACATATATATATAAACAATATA
TTAGGAC
ACCCGCCACAGTAAATTTTCTTGTATTCTCCTAGAAATGAGTTTTAATTTAGTGTGGAAAAAAATAAAAACTTG
CGAATTT
CGAGAAAACAAAACAATTCCTGGTTCAGGACATGTCCATATGTTAACATTCTACTATCACACATGGGGTTGTA
ACAGAAC
TTTTTTGTCATGACTTATAGGGGGGGTATGCATTCAGTTTCACTAATTTTTTTTTTTCCTTGACTCCCGTAAC
TAATAGG
TGCAGCGTTTTCTACTTATAGGAGGGGGCAACCCGAAAATTTTTTTAAAGAGGGGTTGTGGGCCTACTCCAAA
ATTATAA
TGTAATGGGGGCCCTTCCACCCCATTACACCTTCCTTCCTTCCCCTGCTCTGTATCTCTATATCCAGAAATCT
ACAGTCT
AGAACCTTCTCTTCTCCTCTTAGTTTATTCATCCATTTTCTCTAACTCTCTCTATTTAAAAAAATTTGACAAA
AATTTTT
TTTCACCAAGTCGAAAATCCCAGCATATCTACGCTCGTCCCGACTTTGAGAAAAAAAATTTGAAAATTTTGAAA
ATTAAAT

AAGCTGATTTAAAGTATTACAGAAAATATTACAGTCTCGACCCCCCATATGTTTATCAAAATGTTTCATTCAA
ATTACTT
AAAAACTTGTAAATCAGAAGTTAGACTATTTTTTAGTACTTCTCATCCGCTTAGATTTAATTCAAAGGGCGAG
ATTCCAG
ACCATATACATGGGTCTGATGACCCAGGTATTTCCGCCCTTGGATTTTCCATTTTTTTCACTTCTCATCCGCT
TAGATTT
AATTCAAAGGGCGAGATTCCAGACCATATACATGGGTCTGATGACCCAGGTATTTCCGCCCTTGGATTTTCCA
TTTTTTT
CACTTCTCATCCGCTTAGATTTAATTCAAAGGGCGAGATTCCAGACCATATACATGGGTCTGATGACCCAGGT
ATTTCCG
CCCTTGGATTTTCCATTTTTTTCACTTCTCATCCGCTTAGATTTAATTCAAAGGGCGAGAATAACTACGTGGA
ATAAGGA
CGTTGCGGTGGTAGATTTCTCCCTTTTGAAATTTTTGTTTTTTTTCATTTTGGCTAAACTCCGGAGTATAGAC
GTTGTAT
TCATATATATCCTCTCAATTTACAAGTTGGTTTGCTTTTTATCCTTCTAGAAAATTTTCGTCTCGACTTAGAA
AAAAAAA
TTTGGCCAAAAAAAAAACTCTAAGTTCCCAGAAAAATGAACGGCTGGTCAGAATGGCGCGGACAAATACTTTG
AGATGAC
TTATCATAGGGGTTCGTGAAACCTCCACGAAGGTCATCACCCGTTTTGATCAGCCGTTAAAAAATTTTGCTCG
ATTTTTT
TGAAAAATTAGGAGACGTTTCCAAGAGAAAGTACGAGACGCTACTATCGCGGACTTATTGCGTCATATTTATT
TCAATCT
TCTTTTCTCAAAAGGGTTTGAAATAAAAAATGACCAATTTTTCGCTGGAACTCTACGCTTGTACTTTTTTTCT
ACTTAAC
AGAATTGGAGTACGCAGGACTTTAATGATAAGGTCACCAAGGTTTGTGGAGTAGGTGCCCGTCATAAAGTCCT
AGAGATG
CGTGTCGATTTTTGAAACTCAAAATTGCGGGGTTTTATTGGCAAGTTACCAGAGCCACCCTAGGCCTGATGAC
CCAGCTT
TTTTTCCCCTTTTAAATTTTGAAGGCTAGAAACCTGAAAAAAACCTGAAAAAATTGGAGGTGAGATATCATGG
TATAGGT
CATCAGTTCCAAAAACCAACGGTCCTGGAAATGCGTTTCAATTTATTGACCCCATCTCCAGGAAAATTGGCTT
ATGAGAA
TGTGACCCTATACCTTGTGGGATATCAATTTCAATAACCATTTTCAAATCTGCTGTCTGGAAATGTAAAAAAA
ATTTACC
AGGTACTTTCGAATTAGGGGAAATTGACCACATGGACAGCAATATGTATCTATGTGTAATATCAAAAATAATG
GAAATTT
TCCATTTTATAACGGCTTGGATTTAACCCCTATTGTGATAGTAAAAAGAGGACTTGTGTGGGGGTCCTGATGG
AGAAAAT
TTTAAGATTGGAAAAGTCCATTTTTGAAATTTTCTTCGTCAAATTGGAGTTTGGGGAAATCTTACTATCTGAC
ATGTATT
TATATGTCTTCTAGAAAAAATGGGGTTTTAAAATCTGTTAAGTAGAAAGTCAAAAATGACCTTTACTAATTGTC
CGCGGAC
AAATTTTGCGACTCAACGGCTGAGAGTTAAGCAAAGTGATCTTCGTCACCAGGTCACGAATATATACTATTTG
TGATCTA
CCCTGCGATTTTAGACTTTCTGATAGAAAGTCGCTGGGGAAAAAAAAAAAAAAAAATTCGTTCGAATCCATAGC
TCGCACG
CAAGGGCGCTTTTTTAGCACTAGCTCCATGAAGACTTGTCGACTAGATTTTTCCCTTTTTTGAATCTATTTTT
TTTAAAT
TTTGGGTTTTTTCACCCCCTACCCAAATCCCTATACTAGGCCCAACGGGTTTTAGGGGGGTGTACCACAGTCC
CAGCAAG
GGGGTGAAAAAATCCAAAATTTAAAAAAAAATAGATTCAAAAAGGGGAAAAATCTAGTCGACAAGTTTGGGGGG
AGCTAGT
GCTAAAAAAGCGCCCTTGCGTGCGAGCTATGGATTCGAACGAAATGACACTTTTTAACGGCTCATATTTAACT
ATGGTCG

TGAGCCCGGACAAATGCGGTACCACAAGATATGTCGCCAAATATGAATTTTCGGGTCTTCATTTTATTAATCT
AAATTCA
AATTGATTAAGCATGACAGGTCCACCTCGTCGGACAAATAAATATGTCGACCAGAATCTTCCCTTTTACTTCT
AGCTTTA
GCGGTTGGGGAAATTTTCATGGGCCGAGCGTAGTTTCGCTGGGAAAATTTTAAATTTAAATACTTGGATTTAT
CAAAAAT
GATCTTCGTAGCATGGTGTCTTATACCATATGTTTCCCGTCTAGAATAAAAAATTTACCCATGTTATAAAATT
CGAACGA
CCCAAAAAAATTTAACGAGGCCTTATTTCAAGATTCTTTCCAGCGCGCATATGCTCCAAAGTCATGATTTGTCG
TCAAAAA
TTTTTTTTTGATTTTTGTCAAATTTCGTGAAGGAAACGGAATTTTCAAAAACCTACGCTCAAACAGGGTCCCA
TACCCCC
CATATTAGCCTTCAGAAACAACTCTGGAATTGATGGGGAATTGGATTCGAACGAAATTGCGGATTTTTATTTC
CTGAACG
GCCATTCAGGAAGGGTAGACACTCAAACAAACAATAATATATAAGTATGTATGTGGTGTAGAATCTTCCCAAA
GTCCATT
TAGCCCTCTAGAAAATTTTCGTCTCGACTTAGAAAAAAAAATTTTGGGCCAAAAAAAAACTCTAAGTTCCCAG
AAAAATG
AACGGCTGGTCAGAATGGCGCGGACAAATACTTTGAGATGACTTATCATAGGGGTTCGTGAAACCTCCACGAA
GGTCATC
ACCCGTTTTGATCAGCCGTTAAAAAATTTTGCTCGATTTTTTTGAAAAATTAGGAGACGTTTCCAAGAGAAAG
TATGAGA
CGCTACTATCGCGGACTTATTGCGTCATATTTATTTCAATCTTCTTTTCTCAAAAGGGTTTGAAATAAAAAAT
GACCAAT
TTTTCGCTGGAACTCTACGCTTGTACTTTTTTTCTACTTAGTAAAAATATTAAGCGACGGACTTTAATGATAA
GGTCACC
AAGGTTTGTGGAGTAGGTGCCCGTCATAAAGTCCTAGAGATGCGTGTCGATTTTTGAAACTCAAAATTGCGGG
GTTTTAT
TGGCAAGTTACCAGAGCCACCCTAGGCCTGATGACCCAGCTTTTTTTCCCCTTTTAAATTTTGAAGGCTAGAA
ACCTGAA
AAAAACCTGAAAAAAATTGGAGGTGAGATATCATGGTATAGGTCATCAGTTCCAAAAACCAACGGTCCTGGAAA
TGCGTTT
CAATTTATTGACCCCATCTCCAGGAAAATTGGCTTATGAGAATGTGACCCTATACCTTGTGGGATATCAATTT
CAATAAC
CATTTTCAAATCTGCTGTCTGGAAATGTAAAAAAAAATTTACCAGGTACTTTCGAATTAGGGGAAATTGACCAC
ATGGACA
GCAATATGTATCTATGTGTAATATCAAAAATAATGGAAATTTTCCATTTTATAACGGCTTGGATTTAACCCCT
ATTGTGA
TAGTAAAAAGAGGACTTGTGTGGGGGTCCTGATGGAGAAAATTTTAAGATTGGAAAAGTCCATTTTTGAAATT
TTCTTCG
TCAAATTGGAGTTTGGGGAAATCTTACTATCTGACATGTATTTATATGTCTTCTAGAAAAATGGGGTTTTAAA
ATCTGTT
AAGTAGAAAGTCAAAAATGACCTTTACTAATTGTCCGCGGACAAATTTTGCGACTCAACGGCTGAGAGTTAAG
CAAAGTG
ATCTTCGTCACCAGGTCACGAATATATACTATTTGTGATCTACCCTGCGATTTTAGACTTTCTGATAGAAAGT
CGCTGGG
GAAAAAAAAAAAAAAAATTCGTTCGAATCCATAGCTCGCACGCAAGGGCGCTTTTTTAGCACTAGCTCCATGA
AGACTTG
TCGACTAGATTTTTCCCTTTTTTGAATCTATTTTTTTTAAATTTTGGGTTTTTTCACCCCCTACCCAAATCCC
TATACTA
GGCCCAACGGGTTTTAGGGGGGTGTACCACAGTCCCAGCAAGGGGGTGAAAAAATCCAAAATTTAAAAAAAAT
AGATTCA
AAAAGGGGAAAAATCTAGTCGACAAGTTTGGGGGGAGCTAGTGCTAAAAAAGCGCCCTTGCGTGCGAGCTATG
GATTCGA

ACGAAATGACACTTTTTAACGGCTCATATTTAACTATGGTCGTGAGCCCGGACAAATGCGGTACCACAAGATA
TGTCGCC
AAATATGAATTTTCGGGTCTTCATTTTATTAATCTAAATTCAAATTGATTAAGCATGACAGGTCCACCTCGTC
GGACAAA
TAAATATGTCGACCAGAATCTTCCCTTTTACTTCTAGCTTTAGCGGTTGGGGAAATTTTCATGGGCCGAGCGT
AGTTTCG
CTGGGAAAATTTTAAATTTAAATACTTGGATTTATCAAAAATGATCTTCGTAGCATGGTGTCTTATACCATAT
GTTTCCC
GTCTAGAATAAAAAATTTACCCATGTTATAAAATTCGAACGACCCAAAAAATTTAACGAGGCCTTATTTCAAG
ATTCTTT
CCAGCGCGCATATGCTCCAAAGTCATGATTTGTCGTCAAAAATTTTTTTTGATTTTTGTCAAATTTCGTGAA
GGAAACG
GAATTTTCAAAAACCTACGCTCAAACAGGGTCCCATACCCCCCATATTAGCCTTCAGAAACAACTCTGGAATT
GATGGGG
AATTGGATTCGAACGAAATTGCGGACTTTTATTTCCTGAACGGCCATTCAGGAATCCTTTCTAATAACACAGA
AAGTATG
ATACATATACATATATTGTATTTTAATATATTTTTAGTCCAACTTTTTCTTCTAGATTTTTTATAATTCAACC
GTTTTTT
TCGCTGTCCCAACTCCTCAATGGCTGCACTTGGATATAGATTCAAAAGAATAAAGTGGATCGTTATAAAGAAT
AATATTC
AATTATTGGGTCATTATTTAAATTTGATTTATAGAGGGTTTAGAAGAAAAGTAGCGAATATATATATTGCCTT
AATAAAG
ATTTACCATCAATAACATCAATGATTGGCGAAGTACAAAATTTGTACTTTTGATTCAGAGAATTCTAATATGA
AACCTCT
ATCTAGTCATAGCATGTTTATTGCATATTATTAAATATGAAAATTCAAACTGTATTTCAAGGAAAATGATGTT
GGTGCAC
AGGTGACTTATAGGGGGGGTTTCAAACCAAAAGTATAAAAAATTTTCGATTTTAAAAACGGACTTCGTTTCTA
GACGAAA
TGACCTTCGTGGAGCCCCCACAAATATGGGGGGTAGTGACCCAAAAAATGTGATTTTTTCCGAATTTCATTTT
TTGATTT
CATGAACGGCTCGTTTCTAGGCGAAATGATGTTGGTGCACAGGTGACTTATAGGGGGGGTTTCAAACCAAAAG
TATAAAA
AATTTTCGATTTTAAAAACGGACTTCGTTTCTAGACGAAATGACCTTCGTGGAGCCCCCACAAATATGGGGGG
TAGTGAC
CCAAAAAATGTGATTTTTTCCGAATTTCATTTTTTGATTTCATGAACGGCTCGTTTCTAGGCGAAATGATGTT
GGTGCAC
AGGTGACTTATAGGGGGGGTTTCAAACCAAAAATAAAAAATGGGTAAAAATTTTAGGAACGGCTGAGTTTAGG
ATAAGGG
TGTGACGGTGGGATGTGAGAAGTAGTAACTATAGGTATTCAAATACTTGGTAGTTTAAGAGAAAACTTTTCTA
GAGGGTT
TTAGAGAAAAGTAGAAAAATTTACTATCATAATATTTCTTATATATATTCTATATTATTATTAGAACCTCCTA
GAAATGA
GTTTTAATTTAGTGTGGAAAAAATAAAAACTTGCGAATTTCGAGAAAACAAAACAATTCCTGGTTCAGGACAT
GTCCATA
TGTTAACATTCTACTATCACACATGGGGTTGTAACAGAACTTTTTTGTCATGACTTATAGGGGGGGTATGCAT
TCAGTTT
CACTAATATTTTTTTTTCCTTGACTCCCGTAACTAATAGGTGCAGCGTTTTCTACCTATAGGTGGGGTCAGAC
CCACCGA
GGGCCAATGGTAGGGGTGACCCTACCCTATATATTATTATATATATTATATATATATATATAATGGTATATAG
CCTTCGG
TGAGCCTGGCGGCCCTCCGTTAGGCTGCTGGCACTACCCTAGGGTAGCGCGGCCGAAGCGGAACCCACCGCCC
TACCACC
TGGACTAGCTACCCAGGTATAGGGTGGGCCAAACCCTGGTCCTGCTGGTGCCCAGCCACTGGCAGACCGCGCA
GACCGTA

CCTCCCTGTTTACTGCTGTTCAGCTGCTGCACCCCTTAGTGAGGCCGACAGACCCAGCCAGACCCTTCCAGAC
CCTTCCA
ACGAGGCCGACAGACCCAGCCAGACCCTTCCAGACCCTTCCAACGAGGCCGACAGACCCTTCCAGACCCTTCC
AACGAGG
CCGACACGCAGACCGTACCTCCCTGTTTACTGCTGTTCAGCTGCTGCACCCGCTGCACCCCTTAGTGAGGCCG
ACAGACC
CAGCCAGACCCTTCCAGTGAGGCCGACAGACCCAGCCAGACCCTTCCAGACCCTTCCAACGAGGCCGACAGAC
CCAGCCA
GACCCTTCCAGACCCTTCCAACGAGGCCGACAGACCCAGCCAGACCCTTCCATTGCAGCGAACCGACCCAGCC
AGACCCT
TCCATTGCAGCGAACCGACCCAGCCAGACCCTTCCATTGCAGCGAACCGACCCAGCCAGACCCTTCCATTGCA
GCGAACC
GACCCAGCCAGACCCTTCCATTGCAGCGAACCGACCCAGCCAGACCCTTCCATTGCAGTACCCAGCCAGACCC
CTTTCCG
TTTGTGTAGGTTGCGCCAGTGAACCAGGGCCCTCAGGGCGGGTTGGCCCAAAACCCGCGGCTACCGTTGAACC
GATTTTC
GATCGATTCAGCGGCCCAGCCGCACCCCGGGCCTGTGTTTCAGTGATTTAAGGCCTCACAGCCTTGGACCGCA
TTACACG
GCCCACCCTTTTTCAATATTGATTACCTGCGTTAAGCGGGAAACCCTCGAAAGAGGACCTTCATACCTCAGAG
ACCCTAA
CGAATCTATTTATTTAGACTCGAATCGGGTTTAGTACGGCAAAATTTTGGACGGACAGTTAGTAATGGTCTAA
CCATGAA
GGCTAGAAATCCAAAAGGAGCGCAAACAAGGTTAAAAACTCGTTTTGCTCCAGTGA
