# Supplementary material for: An Improved Method for the Isolation of Extrachromosomal DNA from the Pathogenic Free-Living Amoeba Naegleria fowleri
Source: Methods Protoc. 2026 Jul 7;9(4):105. doi: 10.3390/mps9040105 (PMC13398248; doi:10.3390/mps9040105)
Supplement: Supplementary file 1 [file mps-09-00105-s001.zip › mps-4355213-supplementary Figure S1.pdf]

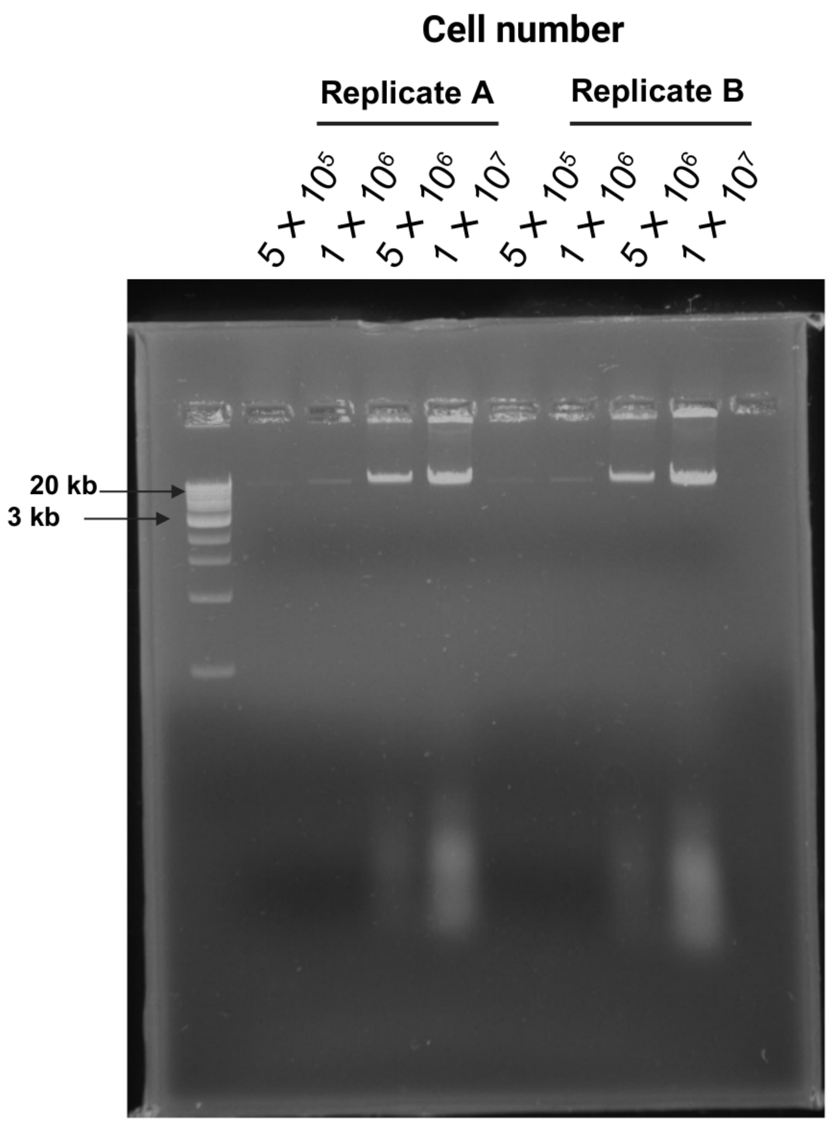
Cell number
Replicate A
Replicate B
5 × 10^5
1 × 10^6
5 × 10^6
1 × 10^7
5 × 10^5
1 × 10^6
5 × 10^6
1 × 10^7
20 kb
3 kb
